# Editorial Notes

**Published:** 1926

**Authors:** 


### Editorial Notes.

**The Journal's New Series.**

WITH this issue the Society's Journal appears in larger pages and type, which it is believed will enhance its value to our readers, in that the matter is presented in a better form, perhaps more worthy of the intimate association of the Medico-Chirurgical Society with the University of Bristol. The gift of the Society's valuable Medical Library to the University, now so splendidly housed, and the allocation of a room for the use of the members of the Society, the accommodation provided for its meetings and many advantages accorded to the Society conferred by the agreement between the University and the Society, but serve to mark their intimate association which we believe will eventuate in still closer ties, promoting the interests of the Medical Faculty and all that it connotes not only within the University but covering its field of influence, which is far and wide.

With the completion of this issue the Editor resigns his charge, with gratitude to the Committee of the Society for appointing him to this office for so many years past, to the members of the Society for their support, and above all to his colleagues on the Editorial Committee. The co-operation of the Assistant Editor, Professor Nixon, throughout these years of the Editor's work, has been a continual encouragement, interrupted only by his foreign service during the war.

While esteeming it a great privilege to have been enabled to initiate this new series, he is confident that under a new Editor the value and sphere of the Journal's work will go forward and become still more widely recognised.

**Oxford D.C.L. conferred on Sir George Wills.**

An interesting ceremony took place at the house of Sir George Wills, Bart., on January 13th, 1926, when the honorary degree of Doctor of Civil Law was conferred on Sir George by the University of Oxford. Oxford and its Colleges have shown, by financial and other support, the greatest interest in Bristol since the foundation of University College. This last token of interest is much appreciated as a distinction conferred on the University of Bristol in the person of its Pro-Chancellor, as well as a richly-deserved tribute to a generous encourager of learning. Sir George was presented to the Vice-Chancellor of Oxford University by Sir Herbert Warren, a friend of boyhood days, who made an appropriate speech in Latin, containing the striking phrase: "Stat hodie domus vel potius palatium Academicum non Athenis non Alexandriâ indignum."

**The Lord Mayor's Hospital Sunday Fund and Addresses by Doctors.**

The following members of the medical profession in Bristol appeared in the pulpits of the churches named to plead the cause of the hospitals on Hospital Sunday, January 31st: Dr. D. A. Alexander, St. James's; Dr. Carey Coombs, Horfield Baptist; Dr. Hubert Bristowe, Brislington Parish Church; Dr. W. A.

Gornall, Eastville United Methodist; Mr. Wilfred Adams, Society of Friends, Redland; Dr. Noel Longley, St. Michael's (City); Dr. R. C. Leonard, Highbury Chapel; Dr. Francis P. Gill, St. Saviour's, Redland; Dr. W. A. L. Holland, St. Anne's, Brislington; Dr. T. B. Dixon, Tyndale Baptist; Dr. E. T. Glenny, Redland Park; Dr. F. J. A. Mayes, Trinity Wesleyan; Dr. Kenneth Wills, Castle Green Congregational; Dr. L. N. Morris, St. Lawrence; Dr. R. S. Carey, Brislington Parish Church.

**The Annual Service for the Medical Profession.**

The Annual Service for the Medical Profession, held on October 18th, 1925, at Bristol Cathedral, was attended by a large and representative gathering of the medical and nursing professions. The preacher was the Right Rev. Dr. E. W. Barnes, Bishop of Birmingham. His text was taken from Ecclesiasticus xxxviii. 1: "Honour a physician with the honour due unto him for the uses which ye may have of him; for the Lord hath created him, and of the Most High cometh healing."

He said that a family physician was often forced by the circumstances of his profession to be a moral guide. In a modern community he, far more than any priest, received confidences and confessions, and if he did not formally grant absolutions, he gave advice by which his patient might win that self-control, that newness of life, which brought peace to the soul. Moreover, a doctor, if he was to maintain the great and honourable traditions of his calling, must preserve a lofty, ethical standard.

They all knew the famous Hippocratic oath, that fine ethical monument of pagan antiquity. The language of that oath, with its sincerity and directness, preserved a fine ideal.

He was happy to think that in this generation men of science were showing a new friendliness towards an enhanced appreciation of the Christian religion. He believed that, in particular, the various branches of the medical profession—and he might surely include nurses under that category — increasingly recognised the value of the spirit of Christ in human affairs, and saw the social dangers associated with the paganism which was, for them, the practical alternative. On the other hand, Christian clergy and ministers were, for the most part, showing a more generous, more open-minded regard for scientific truth of all kinds. Clergy and doctors alike powerfully influenced public opinion, and it would be to the advantage of the whole community if they could learn from and respect one another.

At the present time close co-operation was needed in connection with many social problems which resulted from the changed social conditions of the last hundred years. There was the great problem of the increase of inferior racial stocks, the multiplication of the feeble-minded. Christian sentiment and medical science had together put an end to the ruthless "weeding-out" processes of nature. Could they be content with the result? A solution could only be reached by combining Christian idealism with medical knowledge. But a related, though more difficult question, confronted humanity. He found himself forced to agree with those observers amongst them who traced the ultimate cause of the late war to the extraordinarily rapid increase of European populations during the last century. The great industrial nations instinctively realised that for their safety they must command supplies of food and raw material. The old checks upon over population were pestilence, famine and war. Medical science and social hygiene had eliminated pestilence, international trade had, under normal circumstances, effectively put an end to famine. War remained, and, as the late conflict went far to show, war would destroy their civilisation unless they could make it unnecessary.

So the question confronted them, and would increasingly perplex future generations, how could humanity avoid the danger to civilisation which resulted from excessive population increase? As he saw the future course of human history, that question would become of paramount importance. They must not import into its discussion heated prejudice. Statesmen must make their contributions; from physicians they needed accurate research, physical and psychical. The problem was alike international and personal. He contented himself with pleading that the leaders of Christian opinion and the leaders of medical science must work in cordial co-operation if the great eugenic problems which confronted the whole world, and these overcrowded islands especially, were to be solved.

Dr. Barnes referred to the increase in the number of persons in the community who were mentally defective. There seemed to be no doubt that a tendency to both lunacy and feeble-mindedness was inherited. Christian teachers ought to combine with medical men to protest against the marriage of persons in whom such taints were latent. Knowingly to bring mentally deficient children into the world was a social crime, and the Church ought not to bless a union which in all probability would have that result. Other countries were beginning to take stringent measures to limit the increase of mental defectives. We ought to prepare public opinion to accept similar legislation in our own country. The question was becoming increasingly urgent.—From *Western Daily Press.*

**The Long Fox Lecture.**

The clinical and pathological research related in Dr. Carey Coombs's Long Fox Lecture represents scientific work of great interest and value, a contribution worthy of this foundation in memory of a distinguished Bristol physician, while it is a subject of legitimate pride that this original research emanates from our School. Not long since Dr. Carey Coombs and Dr. C. E. K. Herapath published (in our issue of October, 1924) the article on "Auricular Failure," when *inter alia* Dr. Herapath's work on the sino-auricular node and its import was recorded and illustrated. We may recall also the very early work of Professor Stanley Kent, who till recently held the Chair of Physiology at the Bristol University, for he it was who early in 1892, in his Rolleston Memorial Essay, and again in November,

1892, described the physiological import of the auriculo-ventricular bundle. Although generally spoken of as the auriculo-ventricular bundle of His, it is noteworthy that W. His first published his observations in 1893, the year following Kent's Memorial Prize Essay on the auriculo-ventricular bundle. Earlier still Gaskell (*Journ. of Phys.*, iv., 1883, p. 43) had shown that in lower vertebrates the auricular impulse spread to the ventricles through the muscular bundle connecting them. The clinical import of these observations was not generally recognised till many years later, but in no department of medical science has the last quarter of a century enriched knowledge more than in cardiology.

**Messrs. J. Wright & Sons' Centenary.**

We offer our cordial congratulations to Messrs. John Wright, who have just celebrated their centenary, and wish them the continued success that they have attained so deservedly. From our medical standpoint the evolution of the *Medical Annual* and the authoritative position accorded to this work and to the *British Journal of Surgery* throughout the Empire and the United States of America are outstanding successes of which any publishing house might be proud, while their numerous other publications reflect their energy and artistic effort. This firm has served the medical profession in our city well, and they deserve our thanks. Quite recently they have strengthened their ties with us by a member of our Society being one of the Directorate.

Bristol is well represented in this respect, for our own publishers before many more years pass will have also completed their century of work by successive members of one family to whom our University and the Faculty of Medicine owes much.

---

### Meetings of Societies.

